# General Characteristics and Risk Factors of Cardiovascular Disease among Interstate Bus Drivers

**DOI:** 10.1100/2012/216702

**Published:** 2012-06-04

**Authors:** Raquel Pastréllo Hirata, Luciana Maria Malosa Sampaio, Fernando Sergio Studart Leitão Filho, Alberto Braghiroli, Bruno Balbi, Salvatore Romano, Giuseppe Insalaco, Luis Vicente Franco de Oliveira

**Affiliations:** ^1^Rehabilitation Sciences Master and PhD Program, Nove de Julho University, Avenida Francisco Matarazzo, 612 Agua Branca, 05001-100 Sao Paulo, SP, Brazil; ^2^Department of Medicine, Universidade de Fortaleza, 60811-905 Fortaleza, CE, Brazil; ^3^Division of Pulmonary Rehabilitation, Scientific Institute of Veruno, “Salvatore Maugeri” Foundation, I.R.C.C.S., 28010 Veruno, Italy; ^4^Institute of Biomedicine and Molecular Immunology “A. Monroy,” National Research Council of Italy, 90146 Palermo, Italy

## Abstract

Workers in the transportation industry are at greater risk of an incorrect diet and sedentary behavior. The aim of our study was to characterize a population of professional bus drivers with regard to clinical and demographic variables, lipid profile, and the presence of cardiovascular risk factors. Data from 659 interstate bus drivers collected retrospectively, including anthropometric characteristics, systolic and diastolic blood pressure, lipid profile, fasting blood glucose, meatoscopy, and audiometry. All participants were male, with a mean age of 41.7 ± 6.9 years, weight of 81.4 ± 3.3 kg, and BMI 27.2 ± 3.3 Kg/m^2^; the mean abdominal and neck circumferences were 94.4 ± 8.6 cm and 38.9 ± 2.2  cm; 38.2% of the sample was considered hypertensive; mean HDL cholesterol was 47.9 ± 9.5 mg/dL, mean triglyceride level was 146.3 ± 87.9 mg/dL, and fasting glucose was above 100 mg/dL in 249 subjects (39.1%). Drivers exhibited reduced audiometric hearing at 4–8 kHz, being all sensorineural hearing loss. The clinical characterization of a young male population of interstate bus drivers revealed a high frequency of cardiovascular risk factors, as obesity, hypertension, hyperlipidemia, and hyperglycemia, as well as contributing functional characteristics, such as a low-intensity activity, sedentary behavior, long duration in a sitting position, and high-calorie diet, which lead to excessive weight gain and associated comorbidities.

## 1. Introduction

Obesity has become a global epidemic that has intensified with the availability of low-cost high-calorie foods and an increase in the number of individuals leading to a sedentary lifestyle [1]. Changes have also been occurring in the type of occupation in which workers are engaged, with a move from high-activity to low-activity occupations [2]. Obesity is associated with less participation in work activities, an increase in absenteeism, and a loss of productivity, with a consequent increase in the use of resources [3–5].

Studies in recent decades have demonstrated that workers in the transportation industry are at greater risk of an incorrect diet and sedentary behavior [6, 7]. Bus drivers, in particular, have higher mortality, morbidity, and absenteeism rates due to obesity [8, 9]. Hypertension is one of the main risk factors of this disease [10] and is common among professional drivers [11, 12].

The occupation of driving is also associated with an increased risk of cardiovascular disease [10, 13, 14] and an excessive risk of cerebrovascular disease, such as stroke. The risk factors that contribute to the development of cardiovascular disease are reported in clinical trials carried out in recent decades, including modifiable factors (hypertension, smoking habits, concentrations of HDL and LDL cholesterol, and type 2 diabetes) and nonmodifiable factors (age, gender, and genetic predisposition) [15].

Drivers who carry passengers tend to be at greater risk than those who carry goods [8, 16]. Moreover, behavioral factors among professional drivers contribute considerably to the occurrence of traffic accidents [17, 18]. The World Health Organization estimates that the number of deaths due to traffic accidents will increase by 65% between the years 2000 and 2020, with this figure expected to be as high as 80% in developing countries [18].

The medical and economic costs of traffic accidents are estimated to be 1 to 3% of the gross domestic product of a country (annual cost of, approx. 518 billion dollars). The *Instituto de Pesquisa Econômica Aplicada* (IPEA, Institute of Applied Economic Research) of the Brazilian Federal Government reports that the mean cost of traffic accidents in Brazil is US$ 5,167,000, among which US$ 1,919,000 are spent on victimless accidents, US$ 2,942,000 are spent on accidents that result in injuries, and US$ 2,476,000 are spent on accidents involving deaths [19].

Despite the high costs related to traffic accidents involving professional drivers throughout the world, there are few scientific studies addressing the clinical profile, prevalence of cardiovascular risk factors, and incidence of fatal or incapacitating clinical outcomes (heart failure, coronary disease, cardiovascular and cerebrovascular events, and sleep disordered breathing in this occupation).

The aim of the present study was to characterize a population of professional interstate bus drivers who travel medium and long distances in different work shifts through the assessment of clinical and demographic variables, lipid profile, and the presence of cardiovascular risk factors.

## 2. Methods

### 2.1. Study Design

A retrospective observational study was carried out, involving a population of 659 interstate bus drivers employed by a private bus company in the city of Londrina in province Parana, Brazil. The design, conduction, and divulgation of this study follow the guidelines of the “Strengthening the Reporting of Observational Studies in Epidemiology” STROBE statement for observational studies [20].

### 2.2. Ethical Considerations

This study was carried out in compliance with the principles of Helsinki Declaration and the Guidelines and Regulating Norms for Research Involving Human Subjects formulated by the Brazilian National Health Council of the Ministry of Health in October 1996. The project for this study received approval from the Ethics Committee of the *Universidade Nove de Julho* (Brazil) under process number 329445/2010.

### 2.3. Study Population and Procedures

Data were collected from patient records referring to the last periodic exam of all drivers (January 2010 to January 2011), with the formal consent of the company. Data on anthropometric characteristics (age, weight, height, body mass index (BMI), abdominal circumference and neck circumference), systolic and diastolic blood pressure, lipid profile, fasting blood glucose, meatoscopy, and audiometry were performed.

Blood pressure was measured at rest with the subject remaining seated for 10 minutes. Weight and height were determined using an electronic anthropometric scale (model 200/5, Welmy Industria e Comercio Ltd., Sao Paulo, Brazil) and BMI was calculated using the method stipulated by the World Health Organization [21]. Neck circumference was measured in the region below the laryngeal prominence and abdominal circumference was measured at the height of the iliac crests at the end of expiration; both measurements were performed in anatomic position using a nonelastic metric tape with a precision of 0.1 cm parallel to the ground [22]. The anthropometric measurements were made at the clinic of the Worker Health and Medicine sector of the company by healthcare specialists. Laboratory exams were performed by duly trained nurses at a clinical analysis laboratory, following standard procedures, including the requirement of written informed consent. All biological samples were analyzed by the same clinical analysis laboratory. Audiometry and meatoscopy were performed by a specialized physician in compliance with the guidelines of the American Speech-Language-Hearing Association [23]. Tonal and bone audiometry was performed with a clinical audiometer Welton 1300 (Welton Corporation, Copenhagen, Denmark) by a duly specialized speech and hearing therapist, using the frequencies 250, 500, 1000, 2000, 3000, 4000, 6000, and 8000 Hz. The results were interpreted based on the classification proposed by Merluzzi et al. [24].

### 2.4. Statistical Analysis

The Kolmogorov-Smirnov normality test was used to determine the homogeneity of the population of drivers. Descriptive analysis was then performed, with the results expressed as either mean and standard deviation values or absolute number and percentage, when appropriate. One-way analysis of variance (ANOVA) was performed for comparisons between work shifts, following the confirmation of the homogeneity of the sample. Pearson's correlation coefficients were calculated for the determination of correlations. The SPSS program (version 16.0, Somers, NY, USA) was used for the statistical analysis, considering a 5% significance level and 95% confidence intervals.

## 3. Results

Six hundred fifty-nine employees of a private interstate bus company who travel medium and long distances were involved in the present study. All participants were male, with a mean age of 41.7 ± 6.9 years, weight of 81.4 ± 3.3 kg, and BMI of 27.2 ± 3.3 Kg·m^2^ (Table 1). A total of 353 drivers (53.6%) had worked for up to five years at the firm, 188 drivers (28.5%) had worked six to ten years, 97 drivers (14.7%) had worked 11 to 20 years, and 21 drivers (3.2%) had worked more than 21 years at the firm.

With regard to anthropometric variables, the mean abdominal circumference was 94.4 ± 8.6 cm, with 108 subjects (18.8%) exhibiting values greater than 102 cm. Mean neck circumference was 38.9 ± 2.2 cm, with 40 drivers (6.9%) exhibiting values greater than 40 cm. Based on the BMI, 365 subjects (55.6%) were overweight and 124 subjects (19.6%) were obese (BMI > 30).  Table 2 displays the detailed stratification of the sample based on the criteria of the World Health Organization [21].

**Figure 1:**
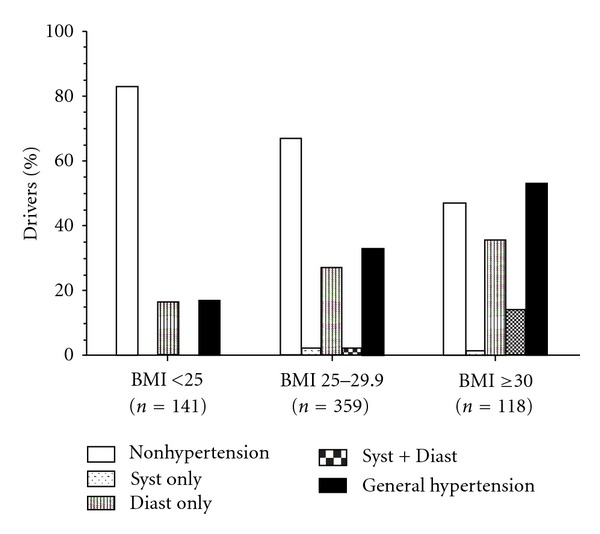
Distribution of population regarding to hypertension stratified for BMI classification. Abbreviations: Syst: systolic; Diast: diastolic; BMI: body mass index.

Based on the criteria stipulated by the Guidelines for the Management of Arterial Hypertension from the European Society of Hypertension and European Society of Cardiology [25], 176 drivers (28.5%) were considered high normal and 41 (6.7%) were hypertensive considering systolic blood pressure. Based on diastolic blood pressure, 194 (31.5%) of the drivers had hypertension (Table 3). In Figure 1, it is demonstrated the distribution of nonhypertensive and hypertensive subjects, according to BMI. The hypertensive subjects are divided into systolic hypertension only, diastolic hypertension only, both systolic and diastolic hypertension, and, finally, general hypertensive subjects.

Mean HDL cholesterol was 47.9 ± 9.5 mg/dL, with 138 subjects (21.7%) exhibiting levels below 40 mg/dL. Mean triglyceride level was 146.3 ± 87.9 mg/dL, with 219 subjects (34.4%) exhibiting levels above 150 mg/dL.  Table 4 displays the total cholesterol values stratified as “desirable,” “borderline,” and “increased,” based on the Third Report of the National Cholesterol Education Program (NCEP) [26]. Fasting glucose was above 100 mg/dL in 249 subjects (39.1%). Based on the “Standards of Medical Care in Diabetes” (2011) [27], 45 subjects (7.1%) were considered prediabetic and 18 (2.8%) were considered diabetic.

One hundred ninety-three drivers (29.5%) worked the dayshift, 222 (33.9%) worked the nightshift, 187 (28.6%) worked the rotating shift, and 52 (8%) were on leave during the data acquisition period. In the comparison of the types of shift, statistically significant differences were found in professional experience at the company. Drivers on the rotating shift had less experience at the company than those on the nightshift (*P* = 0.04) and those who were on leave (*P* = 0.04). Drivers on the rotating shift were older than those on the dayshift (*P* = 0.02) and those on the nightshift (*P* = 0.02). Drivers on leave were older than those on the rotating shift (*P* = 0.001). The BMI of the drivers on leave was higher than that of drivers on the dayshift (*P* = 0.02). Drivers on leave had lower HDL cholesterol values than those on the dayshift (*P* < 0.0001). After confirmation of the *F* statistic, Levene's test revealed that the groups were homogeneous with regard to all other variables.

On the audiometric exam, 56 (8.8%) and 74 subjects (11.7%) exhibited reduced hearing at 4 kHz on the right and left sides, respectively. Reduced hearing was also found at 6 kHz (*n* = 36 (5.7%) on the right side; *n* = 40 (6.3%) on the left side) and 8 kHz (*n* = 26 (4.1%) on the right side; *n* = 27 (4.3%) on the left side). All hearing loss was sensorineural, which is characteristic of noise-induced hearing loss. Regarding hearing complaints among 635 subjects, 51 (7.1%) had some type of clinical hearing complaint, 18 drivers (2.5%) complained of reduced hearing acuity and difficulties understanding speech, and 49 (7.7%) reported ringing in the ears. Moreover, 338 drivers (53.2%) reported exposure to noise in the work environment and 181 (28.5%) reported being exposed to noise outside the work environment. Only 19 (3.3%) made use of earplugs.

## 4. Discussion

The aim of the present study was to characterize a population of interstate bus drivers with regard to demographic and clinical variables, lipid profile, and the presence of cardiovascular risk factors. At the same time, it is suitable to emphasize that this study was not addressed to treat the bus drivers regarding the presence of any cardiovascular risk factors and to verify the impact of these treatments after a followup period.

The population was composed of young male adults, approximately 70% of whom were under 45 years of age (mean age of 41.7 ± 6.9 years). Mean BMI was 27.2 ± 10.7 kg/m^2^, which is characteristic of overweight.

A number of studies have demonstrated the high prevalence of obesity among workers in the transportation industry. According to Moreno et al., this category of workers in Brazil has a higher incidence of obesity, physical inactivity, inadequate diet, smoking habits, high levels of cholesterol and glycemia, hypertension, and obstructive sleep apnea (OSA) in comparison to the general population in Brazil [28, 29]. A study carried out in the United States involving more than 600 thousand workers found the highest prevalence of obesity to be among male employees who work in highway transportation services (31.7%) [30]. A study involving a representative sample of the Australian in productive age compared ten different functional categories with regard to the risk of obesity and found that male employees of the transportation industry had a higher risk of overweight and obesity [31]. In the present study, the prevalence of overweight and obesity was even higher, as more than half of the population of drivers (57.5%) was characterized as overweight and approximately 20% was considered obese, totaling 77.5% of the sample. Similar results are reported in another study involving Brazilian truck drivers, which found prevalence values of 47.8% and 16.2% for overweight and obesity, respectively [32].

Besides increasing cardiovascular risk, obesity in this group of individuals leads to an increase in health costs related to traffic accidents. In a study comparing actual traffic accidents with simulations, Zhu et al. found that obese male drivers demonstrate a substantially greater risk of injury in both situations, especially severe injuries to the upper body, such as the head, face, chest, and spinal column, likely due to the central distribution of fat in this population [33].

Abdominal circumference is a widely used measure for the distribution of fat, as it is indicative of the buildup of visceral adipose tissue or intra-abdominal fat, which, in some cases, may be more harmful than overweight and obesity in general [34]. According to the National Institutes of Health, the cutoff point for abdominal circumference in the male gender is 102 cm [35]. In the present study, the mean value was 94.4 ± 8.6 cm and 108 subjects (18.8%) had values above the cutoff point. Saberi et al. found a 68.3% prevalence of abdominal circumference greater than 102 cm among Iranian drivers, but the study did not differentiate between bus drivers and truck drivers [36]. Another study involving cargo transportation drivers also reports a greater prevalence of an abdominal circumference above the cutoff point (31%) in comparison to the present study [37].

Neck circumference is another factor that merits attention, as this value is reported to correlate better with OSA than BMI. In recent decades, studies have demonstrated that the morphology of the neck in both young adults and older subjects is independently associated with OSA [38–40]. Katz et al. report a mean neck circumference of 43.7 ± 4.5 cm in patients with OSA in comparison to 39.6 ± 4.5 cm in a group of individuals without this condition [41]. Kushida et al. report that a neck circumference equal to or greater than 40 cm is a predictor of OSA with sensitivity and specificity of 61% and 93%, respectively [42]. Parks et al. evaluated 456 bus drivers with a mean neck circumference of 41.5 ± 3.2 cm and those with values of 45.1 ± 2.5 cm were positively screened for OSA [43]. In the present study, mean neck circumference was 38.9 ± 2.2 cm and 40 drivers (6.9%) had a circumference greater than 40 cm.

The prevalence of systemic hypertension was another worrisome finding of the present study. The worldwide prevalence of hypertension is estimated at one billion individuals, with approximately 7.1 million deaths occurring per year due to this condition. According to the World Health Organization systolic blood pressure greater than 115 mmHg accounts for 62% of cases of cardiovascular disease and 49% of cases of ischemic heart disease [44]. Moreover, arterial hypertension is one of the major risk factors of cerebrovascular accident (stroke) [8]. Studies have demonstrated an increase in the prevalence of systemic hypertension among professional drivers [36, 45]. In Brazil, Cavagioni et al. carried out a study on a population of drivers who carry goods and reported a 59% prevalence of systolic blood pressure greater than 130 mmHg or diastolic blood pressure greater than 85 mmHg [37]. In the present study, systolic blood pressure values revealed that more than 1/3 of the bus drivers were characterized as high normal subjects and the diastolic values revealed that 1/3 of the drivers had already systemic hypertension. We have also seen that the greater the BMIs the greater is the proportion of hypertensive subjects.

The sample in the present study had high frequencies of hypercholesterolemia (35.7%) and hypertriglyceridemia (34.4%) associated to overweight/obesity as well as a considerable number of prediabetic subjects. A triglyceride level of 150 mg/dL is one of the five accepted criteria for the definition of the individual risk of cardiovascular disease and type 2 diabetes [26, 46, 47]. Previous studies reported similar findings, such as prevalence rates of 33% and 38% for hypercholesterolemia and hypertriglyceridemia, respectively, among drivers carrying goods [37], and 34.0% and 69.4%, respectively, among drivers carrying passengers [14].

The American Heart Association predicts that the direct and indirect costs of cardiovascular disease in the United States will increase from U$272.5 and U$171.7 billion, respectively, in 2010 to U$818.1 and U$275.8 billion, respectively, in 2030. The majority of these costs are related to short-term and long-term care rather than prevention [48].

From the risk factors that contribute to the development of cardiovascular disease, obesity, hypertension and an increased neck circumference were found in the sample of the present study, which are important risk factors of OSA. This prevalence was also high in other studies with the same population [49, 50].

Prolonged exposure to high-intensity noise was another finding among the drivers. Such exposure causes successive aggression to the internal structures of the ear, such as the organ of Corti, and leads to noise-induced hearing loss (NIHL) [51–53]. In the present study, the prevalence of NIHL was 18.6% and 22.3% in the left and right ears, respectively, and the most accentuated loss was recorded at the frequency of 4 kHz. Correa Filho et al. carried out a study involving drivers from eight different bus companies and found a 32.7% prevalence of NIHL, with a predominance at 6 kHz, which characterizes an advanced stage of NIHL. The authors also found that NIHL was associated to both age above 45 years and longer than six years of work experience [51]. In the present study, mean work experience at the firm was  6.4 ± 5  years. One may hypothesize a possible association between work experience and NIHL based on the continual exposure to external noises and the noise produced by the air through the open window next to the driver's seat during a large portion of the journey.

The work shift is also a risk factor of chronic disease, including cardiovascular disease and metabolic disorders, due to the altered circadian rhythm, changes in lifestyle, tension, and stress at work [54–56].

## 5. Limitations of the Study

The present study has limitations that should be addressed. Our study does not allow the determination of causal associations, since we used a cross-sectional study design, and there was no control group and no followup. Smoking habits were not recorded on the medical records of the drivers; with this information, the classification of cardiovascular risk could have been more reliable. Excessive sleepiness, determined using the Epworth Sleepiness Scale, was another unavailable variable and is known to be a major cause of traffic accidents among professional drivers [57–61].

## 6. Conclusions

The clinical characterization of a young male population of professional interstate bus drivers revealed a high frequency of cardiovascular risk factors, such as obesity, hypertension, hyperlipidemia, and hyperglycemia, as well as contributing functional characteristics, such as a low-intensity activity, sedentary behavior, long duration in a sitting position and high-calorie diet, which lead to excessive weight gain, and associated comorbidities. The high prevalence of obesity associated to hyperlipidemia, diabetes, and hypertension is reason for concern, especially in the population of drivers carrying passengers. We should not forget that the population analyzed in the present study was composed of young adults, which is more worrying. Thus, greater emphasis should be given to the prevention of obesity among individuals in the ideal weight range and those who are overweight. Preventive and educational actions directed at changes in lifestyle with regard to diet and physical activity could be beneficial to this occupational category, which will require prospective studies to verify these outcomes. 

## Figures and Tables

**Table 1:** Anthropometric and clinical characteristics of the population.

Variable	*N*	Mean Value
Age (years)	659	41.7 (6.9)
Weight (Kg)	637	81.4 (1.1)
Height (m)	637	1.73 (0.6)
BMI (Kg/m^2^)	637	27.2 (10.7)
Neck circumference (cm)	578	38.9 (2.2)
Abdominal circumference (cm)	575	94.4 (8.6)
SBP (mmHg)	622	122.1 (10.7)
DBP (mmHg)	622	82.0 (8.3)
HDL (mg/dL)	638	47.9 (9.5)
LDL (mg/dL)	638	111.4 (31.9)
VLDL (mg/dL)	636	28.9 (15.9)
Triglycerides (mg/dL)	638	146.3 (87.9)
Glucose (mg/dL)	637	100.1 (39.1)
Gamma GT (U/L)	635	32.5 (36.5)
Work experience (years)	659	6.4 (5.0)

Description of abbreviations: BMI: body mass index; SBP: systolic blood pressure; DBP: diastolic blood pressure; HDL: high-density lipoprotein, LDL: low-density lipoprotein, VLDL: very low-density lipoprotein.

Data expressed as mean values (standard deviation).

**Table 2:** Classification of the population based on BMI [20, 21].

Classification	Cutoff point	N° (%)
Underweight	<18.5	2 (0.3)
Normal	18.5–24.9	144 (22.7)
Overweight	25–29.9	365 (57.5)
Obesity	≥30	124 (19.5)

Abbreviation: BMI: body mass index.

Data expressed as absolute number (percentage).

**Table 3:** Classification of the population based on systolic and diastolic blood pressure [25].

Classification	Cutpoint	N° (%)
Optimal	SBP < 120	137 (22.1)
Normal	SBP 120–129	264 (42.7)
High normal	SBP 130–139	176 (28.5)
Hypertension	SBP ≥ 140	41 (6.7)

Optimal	DBP < 80	151 (24.5)
Normal	DBP 80–84	256 (41.5)
High normal	DBP 85–89	16 (2.6)
Hypertension	DBP ≥ 90	194 (31.5)

Abbreviations: SBP: systolic blood pressure; DBP: diastolic blood pressure.

Data expressed as absolute number (percentage).

**Table 4:** Distribution of the lipid profile values in the bus drivers population [26].

Classification	Cutpoint (mg/dL)	N° (%)
TC—Desirable	<200	410 (64.3)
TC—Borderline	200–239	160 (25)
TC—Increased	≥240	68 (10.7)

TG—Normal	<150	418 (65,5)
TG—Borderline	150–199	91 (14,3)
TG—Increased	≥200	129 (20,2)

HDL—Desirable	≥40	523 (82)
HDL—Decreased	<40	115 (18)

LDL—Optimal	<100	241 (37,8)
LDL—Desirable	100–129	221 (34,6)
LDL—Borderline	130–159	131 (20,5)
LDL—Increased	≥160	45 (7,1)

VLDL—Desirable	<30	417 (65,6)
VLDL—Borderline	30–40	91 (14,3)
VLDL—Increased	>40	128 (20,1)

Abbreviations: TC: total cholesterol; TG: triglycerides; HDL: high-density lipoprotein, LDL: low-density lipoprotein, VLDL: very low-density lipoprotein.

Data expressed as absolute number (percentage).
